# Cross-Lingual Alzheimer’s Disease Speech Detection: Polarity Inversion and Few-Shot Calibration Strategies

**DOI:** 10.3390/bioengineering13060629

**Published:** 2026-05-27

**Authors:** Qingyi Wang, Meihong Wu

**Affiliations:** 1 School of Informatics, Xiamen University, 422 Siming South Road, Xiamen 361005, China; 37220222203771@stu.xmu.edu.cn; 2 Hearing and Speech Laboratory, Xiamen University, Xiamen 361005, China

**Keywords:** polarity flip, Monte Carlo Polarity Flip Calibration, cross-lingual transfer, few-shot learning, Alzheimer’s disease speech detection

## Abstract

Speech-based non-invasive screening offers a cost-effective and scalable approach for the early detection of Alzheimer’s disease (AD). However, the clinical utility of deep learning models remains severely constrained by the scarcity of labeled speech data in low-resource languages, necessitating cross-lingual transfer learning. Conventional domain adaptation paradigms typically assume semantically consistent feature domains and focus heavily on aligning marginal distributions; however, they suffer catastrophic performance degradation when applied to cross-lingual pathologic speech. By analyzing disease-associated representation vectors within a self-supervised HuBERT space, we uncover a systematic mechanism driving this failure, a phenomenon we term cross-lingual polarity flip, where the direction of disease-relative-to-control feature offsets fundamentally reverses between languages. While prior multilingual studies have largely discarded such dimensional inconsistencies as ungeneralizable noise, a 500-round Monte Carlo stability analysis demonstrates that these flips occur in a highly stable, structural manner across 18.3% of top discriminative dimensions. Leveraging this insight, we introduce Monte Carlo Polarity Flip Calibration (MC-PFC), a few-shot framework designed to explicitly rectify flip orientations. Requiring only five labeled support samples per class from the target domain, MC-PFC robustly estimates direction flips via a separability-weighted ensemble voting mechanism. Evaluated on a strictly held-out Chinese blind test set, MC-PFC achieves an area under the receiver operating characteristic curve (AUC) of 0.871, recovering 99.5% of the performance achieved by a full in-domain trained upper bound (AUC = 0.875). Ablation experiments confirm that direction calibration yields a substantial +0.361 AUC gain, vastly outperforming standard distribution alignment (+0.081). This work establishes a data-efficient paradigm for cross-lingual medical analysis, shifting the clinical AI focus from discarding cross-lingual discrepancies to actively modeling and calibrating them.

## 1. Introduction

### 1.1. Background and Current Landscape

Early screening for Alzheimer’s disease (AD) carries substantial clinical value in delaying cognitive decline. Speech-based non-invasive detection has attracted growing attention due to its low cost and ease of data collection [1,2]. Because labeled medical speech data are distributed highly unevenly across languages [3], transferring discriminative capabilities learned from large-scale English corpora to low-resource languages such as Chinese has become a practical pathway for overcoming this data bottleneck. Recent work has advanced multilingual speech representation learning, exemplified by the VoxPopuli corpus proposed by Wang et al. [4], and has begun to examine the cross-lingual generalizability of AD-related speech features across diverse language pairs [5,6,7].

### 1.2. Research Gap and Hypothesis

When features behave inconsistently across languages, the prevailing paradigm in cross-lingual studies (e.g., [8]) treats the disagreeing dimensions as ungeneralizable noise and discards them. Most domain adaptation methods likewise assume, often implicitly, that cross-domain feature shifts are purely distributional and that the discriminative direction remains invariant. Our preliminary experiments challenge this assumption: a classifier trained on English data and applied directly to the Chinese NCMMSC2021 test set yields an AUC of only 0.510, near chance level, and conventional distribution alignment fails to recover meaningful performance. This observation motivates a central hypothesis: the core cause of cross-lingual transfer failure lies not only in distributional shift, but also in a phenomenon we term cross-lingual polarity flip, whereby the direction of the AD-versus-healthy-control (HC) feature offset reverses on a subset of dimensions between languages.

### 1.3. Contributions and Objectives

Unlike prior work that filters out inconsistent features, this study reframes polarity flips from noise to be discarded into high-value signals to be calibrated. The contributions and objectives are threefold. First, we provide empirical characterization of polarity flips in the self-supervised HuBERT feature space: a 500-round Monte Carlo stability analysis reveals that 11 of the top-60 discriminative dimensions (18.3%) exhibit highly stable flips (flip frequency ≥ 0.70), confirming the structural regularity of the phenomenon. Second, we propose Monte Carlo Polarity Flip Calibration (MC-PFC), a few-shot calibration algorithm that estimates and corrects flip directions under extreme low-resource conditions (only five labeled support samples per class from the target domain) through random sub-sampling and separability-weighted ensemble voting, thereby suppressing estimation variance. Third, we validate the effectiveness of MC-PFC on a 30-subject Chinese blind test set, demonstrating a low-resource cross-lingual transfer scheme that neither discards features nor requires large-scale retraining.

### 1.4. MC-PFC: Direction Calibration Under Few-Shot Conditions

Having identified polarity flips, the next challenge lies in reliably calibrating these flipped dimensions under clinically realistic conditions. In real few-shot scenarios, the target domain typically contains only about ten labeled samples, and a single estimate of the flip direction for each dimension faces extremely high variance: an erroneous polarity calibration directly reverses the classification decision logic, which is more destructive than no calibration at all.

To address this challenge, we propose Monte Carlo Polarity Flip Calibration (MC-PFC). With only K=5 support samples per class from the target domain, MC-PFC estimates flip directions through 500 rounds of random sampling, using support-set separability (the difference between calibrated AD and HC probability means) as weights for weighted ensemble voting, thereby suppressing the variance of small-sample estimates. The method retains the English pretrained classifier and applies only directional corrections to the test-set feature space, requiring no parameter updates to the source model—consistent with the practical deployment requirements of few-shot rapid adaptation.

### 1.5. Main Contributions

The main contributions of this paper are summarized below.

First, at the phenomenon level, we empirically characterize the polarity flip phenomenon in cross-lingual AD detection within the HuBERT feature space, distinguishing it from small-sample estimation noise through a 500-round Monte Carlo stability analysis (18.3% of dimensions exhibit stable flipping). Ablation experiments show that calibrating this phenomenon contributes a +0.361 AUC gain, far exceeding that of distribution shift (+0.081). This result corroborates the cross-lingual feature inconsistencies observed by Lindsay et al. [8] and Pérez-Toro et al. [5], but suggests treating them as a modelable object rather than a filtering target. Second, at the method level, we propose MC-PFC, which achieves stable flip direction calibration with only five support samples per class. On a 30-subject Chinese blind test set, MC-PFC achieves an accuracy of 86.67%, a sensitivity of 80.00%, a specificity of 90.00%, and an AUC of 0.871, comparable to English in-domain performance (AUC = 0.878). Furthermore, MC-PFC recovers 99.5% of the AUC of a full Chinese in-domain upper-bound baseline (AUC = 0.875) trained under the same pipeline, demonstrating that ten labeled support samples can closely approximate the discriminative power of full in-domain training.

The remainder of this paper is organized as follows: Section 2 reviews related work; Section 3 describes the datasets, the feature extraction pipeline, and the design of MC-PFC; Section 4 reports experimental results including the polarity flip discovery, main performance, and ablation analyses; Section 5 discusses the applicability boundaries and limitations of the method; and Section 6 concludes the paper.

## 2. Related Work

### 2.1. Overview of AD Speech Detection Methods

Speech-based AD detection methods can be broadly categorized into three types: methods based on handcrafted acoustic features, methods based on self-supervised pretrained models, and end-to-end deep learning methods. Early work primarily employed handcrafted features such as MFCCs, fundamental frequency, jitter, and pause duration in combination with traditional classifiers such as SVMs [9,10,11]. Self-supervised pretrained models such as Wav2Vec 2.0 [12] and HuBERT [13] learn general-purpose representations through large-scale unsupervised speech pretraining, raising English AD detection accuracy above 85% [14,15], and subsequently being extended by Talkar et al. [6] to pronunciation precision assessment across 12 languages. End-to-end methods directly take raw waveforms or spectrograms as input for joint feature extraction and classification; Chen et al. [16] proposed an improved sampleCNN framework that achieved 86.55% accuracy on the long-audio subset of NCMMSC2021, representing a benchmark result for the Chinese scenario.

### 2.2. Existing Work on Cross-Lingual AD Detection

The ADReSS-M 2023 challenge catalyzed research on the English–Greek language pair [17,18,19,20,21]. Multilingual pretrained resources [4,22,23,24] have provided a foundation for cross-lingual representation learning. Pérez-Toro et al. observed on the English–Spanish pair that temporal features (AUC = 0.75) transfer robustly, while lexico-semantic features (AUC = 0.64) exhibit significantly reduced performance in cross-lingual settings, revealing asymmetric transfer behavior across feature types [5]. Jiang et al. observed cross-lingual consistency of semantic space contraction in the English–Greek pair [7]. Notably, cross-lingual feature inconsistencies have been reported in multiple studies, but are typically treated as non-generalizable noise to be filtered out. The work of Lindsay et al. [8] is the most representative: in English–French bilingual AD detection, they explicitly defined “generalizable features” using the criterion of “significance in both languages,” actively discarded inconsistent dimensions, and observed near-zero overlap in paralinguistic features between the two languages. This paper takes a different perspective: under conditions where only a small number of target-domain labeled samples are available, we investigate whether explicitly calibrating the direction of certain cross-lingually inconsistent dimensions, rather than discarding them, can recover their discriminative contribution—a question warranting empirical investigation.

### 2.3. Limitations of Domain Adaptation Methods in Cross-Lingual AD Scenarios

CORAL [25], MMD [26], and adversarial training [27] have achieved mature applications in image classification and sentiment recognition, but face two common limitations when applied to cross-lingual AD detection. First, these methods rely on large amounts of unlabeled target-domain data, whereas Chinese AD speech data remains scarce. Second, they implicitly assume that cross-domain feature shift directions are consistent and that domain adaptation need only eliminate numerical shifts; our experiments demonstrate that this assumption does not hold in the English–Chinese cross-lingual scenario. Mokgosi et al. [28] proposed a PEFT-based approach for low-resource pathological speech detection that achieved progress, but cross-dataset generalization remains an unresolved challenge.

## 3. Materials and Methods

### 3.1. Datasets

This study uses two datasets: the English ADReSSo 2021 corpus as the source domain and the Chinese NCMMSC2021 corpus as the target domain. Basic statistics are presented in Table 1.

ADReSSo 2021 is derived from the DementiaBank Pitt Corpus [29], with all recordings collected from a standardized Cookie Theft picture description task; each subject typically corresponds to a single recording. NCMMSC2021 encompasses multiple task types, including picture description, verbal fluency tests, and self-introduction, where individual subjects may have multiple recordings. Due to inconsistencies in certain file naming conventions, the effectively aligned test set comprises 30 subjects (10 AD/20 HC), with an HC proportion (67%) substantially higher than that of the training pool (46%); the implications of this class imbalance are discussed in Section 5.3. To ensure an unbiased evaluation, this 30-subject test set was treated as a strictly blind held-out set: it was completely isolated during all phases of model development, including feature extraction, hyperparameter tuning (e.g., the confidence threshold τ and the number of Monte Carlo rounds), and model training. The test set was accessed only once during the final evaluation phase to provide an unbiased reflection of the model’s cross-lingual generalizability.

### 3.2. Feature Extraction

This study employs facebook/hubert-large-ls960-ft (Meta Platforms, Menlo Park, CA, USA) as the acoustic feature extractor [13]. HuBERT learns temporally stable speech representations through self-supervised training based on hidden-unit clustering and masked prediction on raw waveforms. All audio is uniformly resampled to 16 kHz before being fed into the HuBERT encoder; the mean of the last hidden layer across the time dimension yields a 1024-dimensional utterance-level feature vector. For subjects in the Chinese training pool with multiple recordings, all recording-level feature vectors are mean-aggregated to the subject level, ensuring that subsequent evaluation operates at the subject level and thereby avoiding recording-level data leakage.

Feature preprocessing involves two steps. First, *z*-score normalization is applied to all data (English, Chinese training pool, and Chinese test set) using the mean and standard deviation computed from the English training set. Second, a one-way analysis of variance (ANOVA *F*-test, f_classif from scikit-learn v1.6.1) is applied to the English standardized features to rank all 1024 dimensions by AD-versus-HC discriminative power, and the top-60 dimensions (those with the highest *F*-statistics) are selected as the feature subspace for all subsequent experiments. This selection serves two purposes: removing a large number of low signal-to-noise ratio dimensions and mitigating the curse of dimensionality in subsequent small-sample direction estimation. Both the normalization parameters and the dimension selection are derived strictly from the English data alone, with no Chinese data involved in the estimation process—consistent with the unsupervised setting of cross-lingual transfer.

### 3.3. Formal Definition of Polarity Flip

Let the feature means of the AD and HC classes in the source domain (English) training set be $\mu_{\mathrm{AD}}^{\mathrm{EN}}$ and $\mu_{\mathrm{HC}}^{\mathrm{EN}}$, respectively, and define the source domain direction vector $d^{\mathrm{EN}}=\mu_{\mathrm{AD}}^{\mathrm{EN}}-\mu_{\mathrm{HC}}^{\mathrm{EN}}\in R^{60}$. The sign of the *i*-th component reflects the discriminative direction of the *i*-th feature dimension in the English scenario: if $d_{i}^{\mathrm{EN}}>0$, the AD group mean exceeds that of HC on this dimension, and the classifier treats “higher values on this dimension” as evidence for AD.

Similarly, for a target domain (Chinese) support set $S=S_{\mathrm{AD}}\cup S_{\mathrm{HC}}$ ($|S_{\mathrm{AD}}\left|\;=\;\right|S_{\mathrm{HC}}|\;=K$), define the target domain direction vector $d^{\mathrm{ZH}}\left(S\right)={\bar{x}}_{S_{\mathrm{AD}}}-{\bar{x}}_{S_{\mathrm{HC}}}$. The polarity flip on dimension *i* is defined as

(1) $${\mathrm{flip}}_{i}\left(S\right)=⊮\;\left[\;\mathrm{sign}\left(d_{i}^{\mathrm{EN}}\right)\neq \mathrm{sign}\left(d_{i}^{\mathrm{ZH}}\left(S\right)\right)\;\right]$$

where ⊮[·] denotes the indicator function. When ${\mathrm{flip}}_{i}=1$, the discriminative direction of the *i*-th feature in the target domain is opposite to that in the source domain; if the English-trained classifier is directly applied to features on this dimension, its contribution changes from a correct discriminative signal to a misleading interference. The goal of polarity calibration is to identify these flipped dimensions and negate the corresponding values in the test samples, thereby aligning the discriminative directions of the target domain feature space with those of the source domain.

### 3.4. MC-PFC Algorithm

#### 3.4.1. Single-Round Calibration Procedure

With only a small number of target-domain support samples available, a single estimate of $d^{\mathrm{ZH}}\left(S\right)$ exhibits high variance, rendering any single flip determination unreliable. MC-PFC addresses this issue by suppressing variance through Monte Carlo ensembling. The single-round calibration procedure (episode) proceeds as follows:

Step 1: Support set sampling: K=5 AD subjects and K=5 HC subjects are randomly drawn from the Chinese training pool to form the current round’s support set $S^{\left(t\right)}$.

Step 2: Direction estimation and flip determination: The target direction vector $d^{\mathrm{ZH}}\left(S^{\left(t\right)}\right)$ is computed and compared sign-wise with $d^{\mathrm{EN}}$ to obtain the current round’s flip mask $m^{\left(t\right)}\in {\left\{-1,+1\right\}}^{60}$, where flipped dimensions take the value −1 and stable dimensions take +1.

Step 3: Polarity correction: The correction ${\tilde{X}}^{\left(t\right)}=X_{\mathrm{test}}⊙m^{\left(t\right)}$ is applied to all test samples, where ⊙ denotes element-wise multiplication. Only sign flipping is performed; no mean shifting is applied.

Step 4: Test sample prediction: The corrected features ${\tilde{X}}^{\left(t\right)}$ are fed into the English pretrained RBF-SVM (C=10.0, class-balanced) to obtain the current round’s AD probability estimates $p^{\left(t\right)}\in {\left[0,1\right]}^{30}$.

Step 5: Dynamic threshold correction: The support set $S^{\left(t\right)}$ undergoes the same $m^{\left(t\right)}$ correction and is then passed through the same SVM to obtain AD probability predictions for each support sample. The probability center of the current round, $\theta^{\left(t\right)}$, is computed as the mean of the median predicted probabilities of $S_{\mathrm{AD}}^{\left(t\right)}$ and $S_{\mathrm{HC}}^{\left(t\right)}$. Both the support and test sample probabilities are then shifted to align the center of the probability distribution to 0.5: $p^{\left(t\right)}\leftarrow \mathrm{clip}\left(p^{\left(t\right)}+0.5-\theta^{\left(t\right)},\;0,\;1\right)$.

#### 3.4.2. Monte Carlo Ensembling and Separability Weighting

The above procedure is repeated for T=500 rounds, yielding ${\left\{p^{\left(t\right)}\right\}}_{t=1}^{T}$. Different rounds exhibit quality variation in their support sets: when a support set happens to include typical AD and HC samples, the direction estimate is more accurate; otherwise, the bias is larger. To account for this variation, we define the separability of the current round as $\delta^{\left(t\right)}=\left|{\bar{p}}_{S_{\mathrm{AD}}}^{\left(t\right)}-{\bar{p}}_{S_{\mathrm{HC}}}^{\left(t\right)}\right|$, that is, the absolute difference between the mean calibrated predicted probabilities of the AD and HC groups in the support set. A higher separability value indicates better AD/HC distinguishability in the calibrated feature space for that round, and thus a more reliable estimate.

The final predicted probability is computed as the weighted average over all rounds:

(2) $$\hat{p}=\frac{\sum_{t=1}^{T}{\left(\delta^{\left(t\right)}\right)}^{2}\cdot p^{\left(t\right)}}{\sum_{t=1}^{T}{\left(\delta^{\left(t\right)}\right)}^{2}}$$

The weights are squared separability values, which amplify the influence of high-quality rounds and further suppress noise from low-quality rounds. The final decision compares $\hat{p}$ against the decision threshold τ=0.55: samples above the threshold are classified as AD and those below as HC. The choice of τ=0.55 is motivated by Bayesian decision theory under the class prior imbalance of the test set (AD:HC =1:2); when the negative class dominates, the optimal decision boundary should shift moderately toward higher probabilities to control the false positive rate (see Section 4.3.3 for threshold sensitivity verification).

To assess the robustness of the method to randomness, the entire procedure is independently executed with five fixed random seeds, and the results are reported as the mean across all five runs; convergence behavior is discussed in Section 4.2 and Section 5.3.

#### 3.4.3. Rationale for Design Decisions

MC-PFC departs from more intuitive approaches in two key design choices. First, it performs only polarity flipping without distribution shifting. A natural extension would be to first shift the Chinese features toward the English mean using support set statistics and then apply polarity flip calibration. However, ablation experiments (Section 4.3) show that this combination actually degrades performance relative to polarity flipping alone because the support set mean estimate itself exhibits high variance at K=5 and the resulting shifting noise disrupts the local structure of the feature space, interfering with subsequent flip determination.

Second, separability weighting is used rather than equal-weight voting. Across 500 rounds of sampling, the quality of flip estimates varies substantially; equal-weight voting allows for low-quality rounds to dilute contributions from high-quality rounds. Separability weighting employs the post-calibration AD/HC distinguishability of each round in the support set as a proxy for reliability, retrospectively assessing the quality of each round and reducing ensemble noise without requiring additional support samples.

## 4. Experimental Results

### 4.1. Direct Transfer Failure and Empirical Evidence of Polarity Flipping

**Figure 1:**
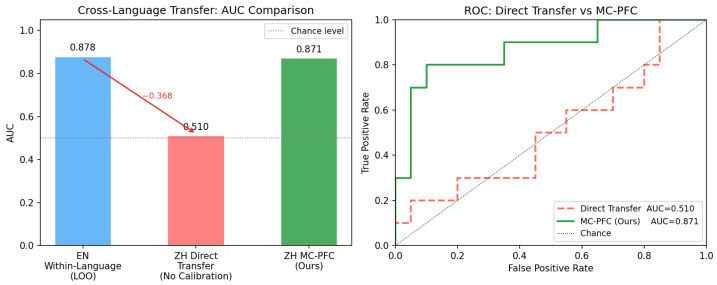
Cross-language transfer: AUC comparison (**left**) and ROC curves (**right**). The English in-domain classifier (AUC =0.878) drops to chance level (AUC =0.510) upon direct transfer to Chinese; MC-PFC recovers performance to AUC =0.871.

To localize the specific mechanism of cross-lingual transfer failure, we first establish baseline performance within the source domain and then observe the performance drop upon direct transfer to the target domain. Using leave-one-out cross-validation (LOOCV) on the ADReSSo 2021 English data, HuBERT-SVM achieves an in-domain AUC of 0.878, confirming that the feature–classifier combination possesses strong discriminative power within the source domain. When the same classifier is applied to the 30-subject Chinese blind test set without any target-domain adaptation, the AUC drops to 0.510—equivalent to chance-level performance. Figure 1 visualizes this collapse: AUC falls by 0.368 from the English in-domain level (0.878) to near chance, and the corresponding ROC curve confirms that the discriminative ability of the classifier is effectively lost upon direct transfer.

Notably, under the no-calibration condition, the system exhibits a sensitivity of 70.00% but a specificity of only 30.00%; the classifier tends to label most samples as AD. This systematic bias toward AD suggests that the problem extends beyond mere numerical distribution shift: if the discriminative directions of certain dimensions are being used in reverse, the classifier would systematically misclassify HC subjects as AD on those dimensions, which is consistent with the observed behavior.

To quantitatively examine this hypothesis, we performed 500 rounds of Monte Carlo support set sampling (K=5 per round) on the Chinese training pool following the formalization in Section 3.3 and tabulated the flip frequency distribution of the top-60 HuBERT feature dimensions. The results are presented in Table 2: 11 of 60 dimensions (18.3%) exhibit highly stable flipping with a flip frequency ≥0.70, another 11 dimensions (18.3%) show systematic stability with a flip frequency ≤0.30, and the remaining 38 dimensions (63.4%) fall between these two extremes.

**Figure 2:**
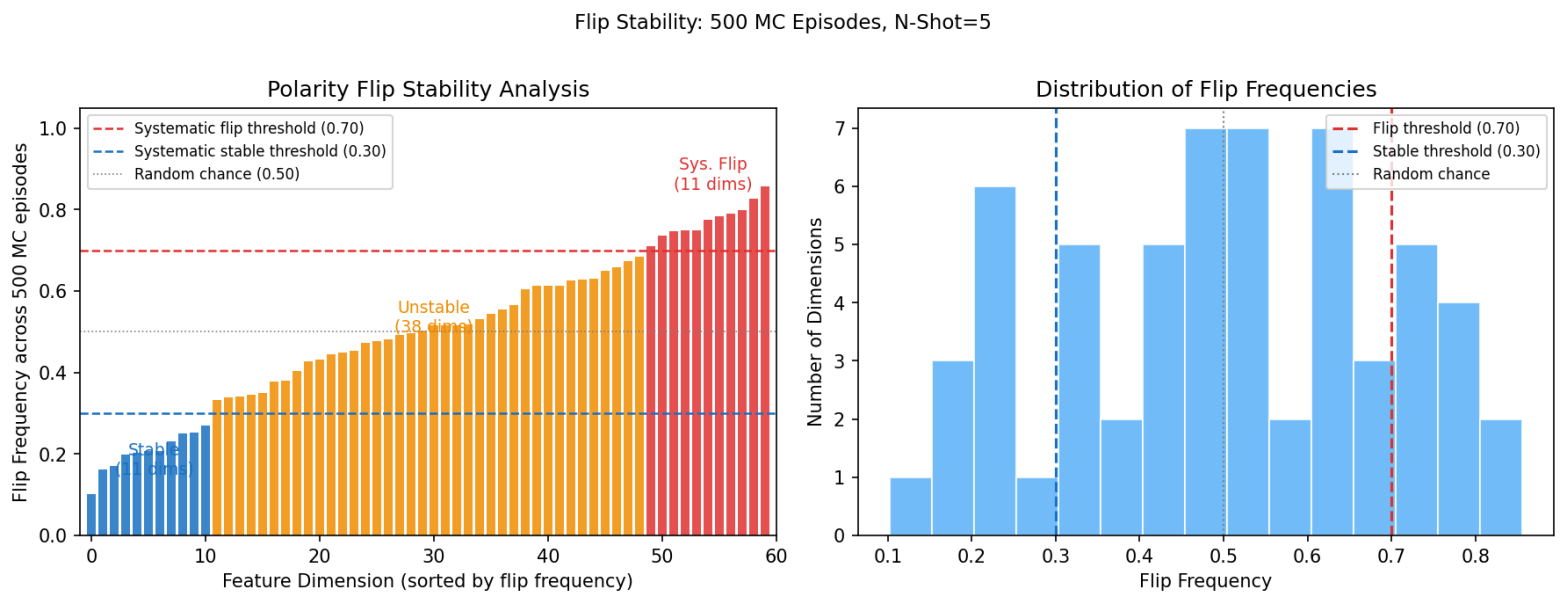
Polarity flip stability analysis (500 MC episodes, K=5). (**Left**): Flip frequency per dimension sorted in ascending order, with thresholds for systematic stability (0.30, blue dashed) and systematic flipping (0.70, red dashed). (**Right**): Histogram of flip frequencies across all 60 dimensions.

The overall distribution of flip frequencies is not concentrated around 0.5 (Figure 2), indicating that the flip behavior of individual dimensions cannot be entirely attributed to small-sample noise; a subset of dimensions (approximately 18.3%) consistently exhibit directions opposite to those observed in English across multiple resamplings. This result corroborates the cross-lingual feature inconsistencies reported by Lindsay et al. [8] and Pérez-Toro et al. [5]: there indeed exist dimensions in cross-lingual settings whose discriminative behavior cannot be simply subsumed under the “distribution drift” framework. The key distinction is that we do not discard these dimensions as non-generalizable noise.

**Figure 3:**
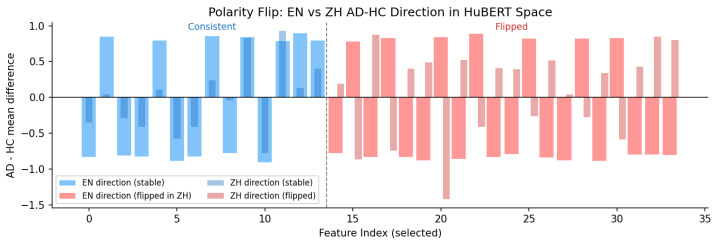
Polarity flip visualization: EN vs. ZH AD–HC direction in HuBERT space. Blue bars indicate dimensions with consistent direction across languages; red/pink bars indicate flipped dimensions where the EN and ZH direction vectors have opposite signs.

Figure 3 further illustrates the polarity flip phenomenon at the dimension level, displaying the AD-minus-HC mean difference for selected dimensions in both English and Chinese. On the left (blue) side, stable dimensions show consistent directions between languages; on the right (red) side, flipped dimensions exhibit EN and ZH direction vectors with opposite signs, visually confirming the directional reversal.

### 4.2. Main Results of MC-PFC

Applying MC-PFC (Section 3.4) to the 30-subject Chinese blind test set yields the results shown in Table 3. The system achieves an accuracy of 86.67%, a sensitivity of 80.00%, a specificity of 90.00%, and an AUC of 0.871. Compared to the English in-domain AUC of 0.878, the difference is only 0.007, indicating that polarity flip calibration substantially recovers cross-lingual discriminative power without noticeably compromising the source-domain advantage.

**Figure 4:**
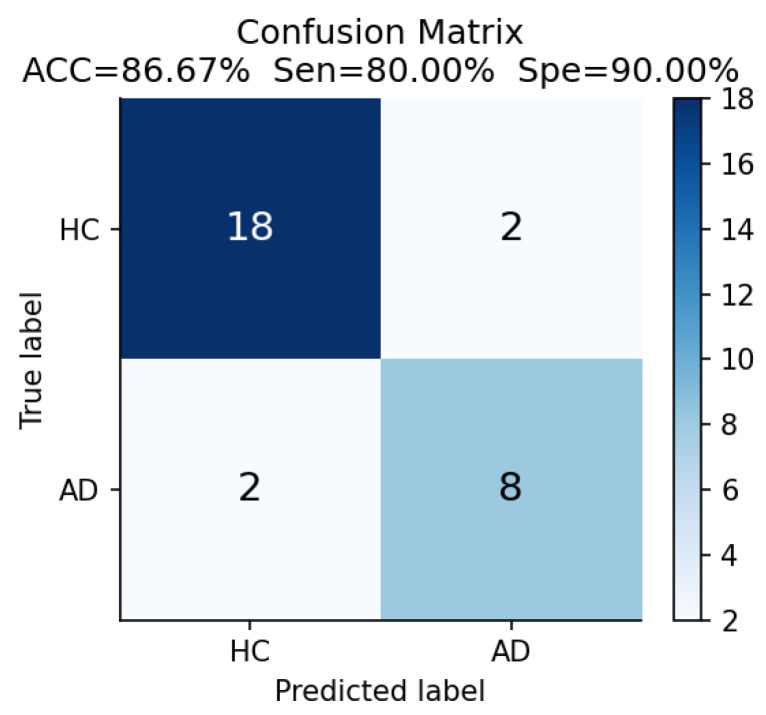
Confusion matrix of MC-PFC on the 30-subject Chinese blind test set. Accuracy =86.67%; Sensitivity =80.00%; Specificity =90.00%.

The confusion matrix (Figure 4) shows that the system correctly identifies eight AD subjects (true positives) and eighteen HC subjects (true negatives) out of thirty, with two false negatives and two false positives. In a clinical screening context, 80% sensitivity implies that approximately two out of every ten AD patients face missed-diagnosis risk, while 90% specificity corresponds to a low false-alarm rate. It should be noted that, with only ten AD subjects in the test set, a single prediction error shifts sensitivity by approximately ten percentage points; sample size limitations are further discussed in Section 5.3. To formally quantify this uncertainty, we apply non-parametric percentile bootstrap (1000 iterations): the observed ACC of 86.67% corresponds to a 95% CI of [73.3%, 96.7%], and the F1 score of 79.0% corresponds to a 95% CI of [53.3%, 95.7%].

Five fixed seeds yield identical results (standard deviation of zero across all metrics), confirming that the 500-round Monte Carlo ensemble has fully converged on the 30-subject test set.

To further contextualize the performance of MC-PFC, we trained a same-configuration HuBERT-SVM on all 70 subjects from the Chinese training pool as an in-domain upper-bound baseline. This baseline achieves an AUC of 0.875, nearly identical to that of MC-PFC (AUC =0.871)—a recovery ratio of 99.5%—indicating that only ten support samples combined with polarity flip calibration can closely approximate the discriminative power of full in-domain training. Interestingly, the accuracy of the upper-bound baseline (76.67%) is actually lower than that of MC-PFC (86.67%). This discrepancy arises because the training set and test set have inconsistent class priors (training AD:HC ≈1:1, test 1:2), rendering the fixed 0.5 threshold suboptimal on the test set, whereas the dynamic threshold correction step of MC-PFC (Section 3.4.1, Step 5) implicitly compensates for this shift.

As an additional reference, Chen et al. [16] reported an accuracy of 86.55% on the long-audio subset of NCMMSC2021 using the full Chinese training set with an end-to-end framework. The accuracy of our method on the 30-subject blind test set (86.67%) falls in the same numerical range. However, the evaluation protocols differ: our evaluation operates at the speaker level with mixed long and short audio, whereas that of Chen et al. [16] uses utterance-level evaluation with long and short audio reported separately. Consequently, a strict performance ranking is not warranted. This numerical comparison serves only as a positioning reference, suggesting that, with an appropriate transfer mechanism, a small number of target-domain labeled samples may approximate the detection capability of full in-domain training, although this conclusion requires further verification under unified evaluation protocols.

### 4.3. Ablation Experiments

To disentangle the sources of performance gains in MC-PFC, we evaluate four calibration strategy configurations under a unified MC framework (500 rounds, K=5, five seeds) together with two alternative feature space configurations. The results are presented in Table 4 and Table 5.

#### 4.3.1. Calibration Strategy Ablation

Limited contribution of distribution shift. Distribution shift alone raises AUC from 0.510 to 0.591 and specificity from 30.00% to 55.00%. The improvement is modest, and the AUC remains far below clinically usable levels. This result suggests that the large-scale pretraining of HuBERT has already implicitly aligned the overall distributions of the English and Chinese datasets to some extent; additional shifting based on means estimated from only five support samples likely introduces estimation noise that partially offsets potential gains.

Polarity flip calibration is the primary source of performance recovery. Polarity flipping alone raises AUC substantially to 0.871 (a gain of +0.361), representing the best performance among all four configurations. Compared to the +0.081 gain from distribution shift, the contribution of polarity flip calibration is markedly larger. This contrast is consistent with the observation in Section 4.1 that the system under no calibration exhibits a systematic bias toward AD classification: once the 18.3% of stably flipped dimensions have their signs explicitly corrected, the decision direction of the classifier is restored, and specificity increases from 30.00% to 90.00%.

**Figure 5:**
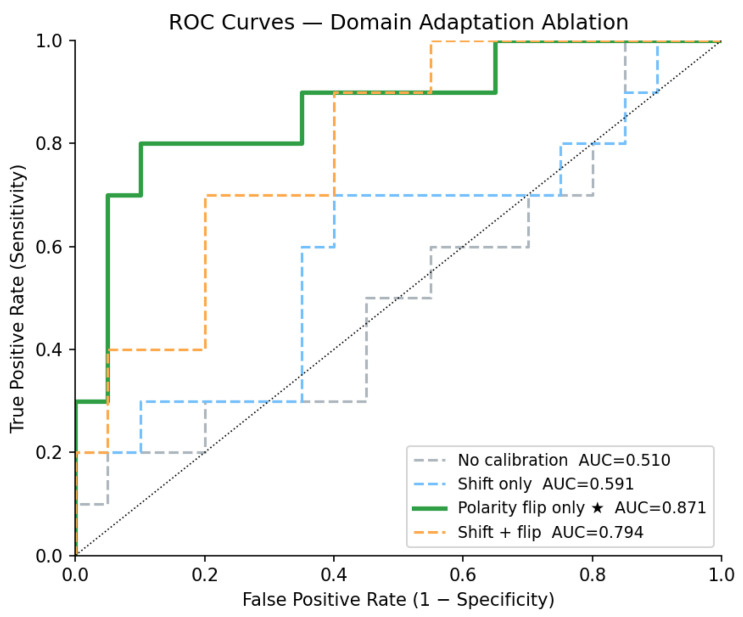
ROC curves for the four calibration strategy ablation configurations. The polarity-flip-only configuration (solid green; ★ denotes the best-performing configuration) dominates all other configurations across the full operating range.

Counter-intuitive degradation of the combined strategy. Intuitively, the combination of shift and flip should outperform either strategy in isolation, yet the measured AUC (0.794) is actually lower than that of flip-only (0.871). A plausible explanation for this is that the mean estimate derived from five support samples has high variance, and the numerical perturbation introduced by shifting affects the accuracy of subsequent flip direction determination, causing the flip mask on certain dimensions to be erroneous. The ROC curves for all four configurations are shown in Figure 5.

#### 4.3.2. Feature Space Ablation

ComParE handcrafted features + MC-PFC (Configuration 5). ComParE 2016, extracted using openSMILE v2.6.0 (audEERING GmbH, Gilching, Germany), is a standardized handcrafted feature set widely used in AD detection, covering 6373 acoustic descriptors. After keyword-based blacklist filtering to remove pitch, jitter, and other tone-related dimensions that strongly interfere between Chinese and English, approximately 6056 effective dimensions remain; the top-15 dimensions ranked by English single-dimension AUC are used as input. Under the identical MC-PFC framework used for the HuBERT experiment, the ComParE feature stream achieves an AUC of only 0.455—below chance level. This result demonstrates that when feature dimensionality is excessively high and the per-dimension signal-to-noise ratio is limited, K=5 support samples are insufficient to support stable direction estimation; single-round direction determination errors dominate, and ensemble voting cannot compensate. The effectiveness of MC-PFC depends on a feature space with high signal-to-noise ratio and controlled dimensionality; the HuBERT top-60 subspace satisfies this condition, whereas ComParE does not.

Semantic perplexity single stream (Configuration 6): Log perplexity features extracted via Whisper-base (OpenAI, San Francisco, CA, USA) [30,31] + GPT-2 (uer/gpt2-chinese-cluecorpussmall, UER, accessed via Hugging Face) transcription achieve an AUC of 0.620 as a standalone stream—above chance, but substantially below the 0.871 achieved with HuBERT. This confirms that, in the current pipeline, deep acoustic representations from HuBERT provide the primary discriminative power for cross-lingual AD detection. Combining Configurations 1–6, a clear conclusion emerges: MC-PFC is effective on the HuBERT top-60 subspace because this feature space simultaneously satisfies three conditions—high signal-to-noise ratio, controlled dimensionality, and broad acoustic coverage from large-scale pretraining that yields compact, discriminative representations even for unseen languages. Directly applying this method to other feature spaces may not yield comparable results.

#### 4.3.3. Threshold Sensitivity

**Figure 6:**
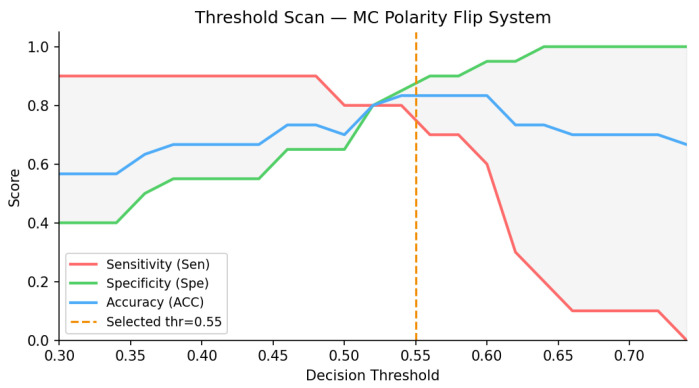
Threshold sensitivity analysis. Horizontal axis: decision threshold τ; curves show Sensitivity (red), Specificity (green), and Accuracy (blue). The selected threshold τ=0.55 (orange dashed line) falls within a broad plateau of peak accuracy.

The final decision threshold τ=0.55 is chosen based on Bayesian analysis under the class prior imbalance of the test set (AD:HC =1:2)—when the negative class dominates, the optimal decision boundary should shift moderately toward higher probabilities to control the false positive rate. To verify the robustness of this choice, we evaluate system performance across τ∈[0.30,0.74]. Within approximately τ∈[0.53,0.60], accuracy remains stable at the peak level of 86.67%, exhibiting a flat-top distribution (Figure 6), which indicates that performance is insensitive to the threshold and the final result does not depend on precise threshold selection.

## 5. Discussion

### 5.1. Interpretation of Key Results and Their Engineering and Biomedical Implications

The central finding of this study is that calibrating only the polarity-flipped feature dimensions in English-to-Chinese cross-lingual AD speech detection recovers the model’s AUC from 0.510 to 0.871. The magnitude of this gain (+0.361) substantially exceeds the improvement obtained by distribution shift correction alone (+0.081).

From an engineering perspective, this result demonstrates the superiority of direction calibration over distribution alignment in the few-shot transfer setting. When only a handful of target-domain samples are available, forcing global distribution alignment in a high-dimensional space tends to introduce estimation noise, whereas binary polarity correction on selected dimensions restores the source-domain discriminative power with considerably higher robustness.

From a biomedical and phonetic perspective, the polarity flip phenomenon reveals an asymmetric expression of pathological speech features under different linguistic systems. Mandarin, as a tonal language, imposes strict phonemic constraints on pitch and fundamental frequency, while English, as a stress-timed language, governs prosody through fundamentally different rules. Neurodegenerative changes associated with AD, such as deteriorating articulatory motor control and delayed lexical retrieval, are filtered through these two distinct linguistic production systems and, consequently, project group-level differences in opposite directions along certain temporal or acoustic representation dimensions. This linguistic asymmetry in pathological speech expression has also been noted in broader calls for language-inclusive clinical research [32].

### 5.2. Comparison with Existing Literature and Positioning

Previous studies (e.g., [5,8]) have repeatedly observed cross-lingual feature inconsistencies, yet the dominant paradigm in the field treats such features as ungeneralizable noise and discards them. The distinctive contribution of this work lies in redefining the 18.3% of stably flipped dimensions from noise to be filtered into high-value signals to be calibrated.

Our results show that, after explicit direction calibration, a transfer model relying on only ten labeled samples achieves an accuracy of 86.67%, approaching the numerical range of an end-to-end baseline trained on the full Chinese training set (86.55%). This finding offers the field of cross-lingual pathological speech analysis a new perspective that moves from feature discarding toward feature retention and correction.

### 5.3. Limitations and Their Potential Impact on Conclusions

Despite the effectiveness demonstrated by MC-PFC, several limitations warrant discussion.

First, constrained by the available aligned data, the target-domain blind test set contains only 30 subjects with a class imbalance of 10 AD to 20 HC. The small positive-class base causes the sensitivity metric to fluctuate by approximately ten percentage points with a single sample prediction change, limiting the statistical stability of the results when projected onto larger clinical populations.

Second, the success of MC-PFC depends heavily on the compactness and implicit multilingual alignment of the underlying feature space such as HuBERT. Ablation experiments show that the method fails entirely on high-dimensional handcrafted acoustic features such as ComParE, with the AUC dropping to 0.455 due to the curse of dimensionality. Moreover, the deep self-supervised representations of HuBERT lack intuitive physical interpretability, making it difficult to explain to clinicians the specific phonetic mechanisms that give rise to the observed flips.

Third, in Mandarin, fundamental frequency and pitch variation carry core semantic distinctions, whereas in English, prosodic features primarily serve syntactic structuring and focus marking. The pathological prosodic degradation caused by AD interacts with tonal constraints in Mandarin and stress patterns in English in fundamentally different ways, producing divergent acoustic manifestations. This difference in underlying linguistic mechanisms constitutes a potential confounding factor. Although the present work empirically identifies and calibrates the flipped dimensions through algorithmic means, it does not yet disentangle, at the physical level, the deep interaction between pathological degradation and language-specific prosodic rules.

### 5.4. Future Research Directions

Building on the findings and limitations discussed above, future work can extend in several directions.

A first direction is to validate the systematicity and structural regularity of polarity flips on larger independent clinical datasets and across additional language family pairs, such as other languages within the Indo-European and Sino-Tibetan families, in order to establish the boundaries of its generalizability.

A second direction involves combining explainable machine learning techniques with the HuBERT representation space to map the specific flipped dimensions onto acoustically grounded physiological features such as vowel formant dynamics, articulatory perturbation, and pause patterns, thereby providing solid pathological-phonetic evidence for the polarity flip phenomenon.

A third direction concerns removing the current dependence on a small number of labeled target-domain support samples. Future research may explore self-supervised learning or feature-clustering distributions to achieve zero-shot or fully unsupervised estimation of flip directions without any target-domain labels. Existing few-shot learning frameworks such as prototypical networks [33] and model-agnostic meta-learning [34] may also provide useful inductive biases for this task.

A fourth direction addresses task heterogeneity. The English source-domain dataset used in this study (ADReSSo 2021) employs only the Cookie Theft picture description task, whereas the Chinese target-domain dataset (NCMMSC2021) encompasses multiple task types including spontaneous narrative, reading, and cognitive question answering. The cognitive load and speech production mechanisms elicited by different tasks exhibit substantial heterogeneity. Future studies should rigorously control for task effects by designing cross-lingual experiments with homogeneous tasks, thereby eliminating task-induced bias and further strengthening the generalization robustness of the calibration model.

## 6. Conclusions

This paper addresses the problem of transferring English-trained AD speech detection models to the Chinese scenario. We attribute direct transfer failure to the cross-lingual reversal of discriminative directions on certain dimensions of the HuBERT feature space—a phenomenon we term *cross-lingual polarity flip*. A 500-round Monte Carlo stability analysis shows that 18.3% of the top-60 discriminative dimensions exhibit highly stable flipping that is not attributable to small-sample noise. Based on this finding, we propose MC-PFC, which suppresses estimation variance through Monte Carlo ensembling and separability weighting with only five support samples per class. On a 30-subject Chinese blind test set, MC-PFC achieves an accuracy of 86.67% and an AUC of 0.871, comparable to English in-domain performance (AUC =0.878) and recovering 99.5% of the full Chinese in-domain upper-bound AUC (0.875). Ablation experiments confirm that polarity flip calibration contributes a +0.361 AUC gain, far exceeding that of distribution shift (+0.081), and combining the two strategies actually degrades performance—establishing polarity flip calibration as the dominant mechanism for cross-lingual transfer recovery in this scenario. It should be noted that the current conclusions rest on a 30-subject blind test set and a single language pair (English–Chinese), with limited statistical power; our numerical comparison with the Chinese in-domain end-to-end baseline does not constitute a strict ranking due to differences in evaluation protocols. Validation under larger-scale, multi-language-pair, and unified evaluation protocol conditions represents the core direction for future work. The central claim of this paper can be summarized as follows: in cross-lingual pathological speech transfer, the consistency of discriminative directions constitutes a dimension independent of distribution alignment that warrants explicit examination and calibration. Recent work leveraging large language models for AD detection [35] further underscores the rapid evolution of this field, within which the present direction-calibration paradigm offers a complementary and orthogonal contribution.

## Figures and Tables

**Table 1:** Experimental datasets.

Dataset	Language	Total Subjects	AD/HC	Role
ADReSSo 2021	English	166	87/79	Source domain (training)
NCMMSC2021 training pool	Chinese	70	38/32	Support set sampling pool
NCMMSC2021 test set	Chinese	30	10/20	Blind test set

**Table 2:** Flip stability statistics (500 MC rounds, top-60 dimensions).

Category	Criterion	Dimensions	Proportion
Systematic flipping	Flip frequency ≥ 0.70	11	18.3%
Unstable	0.30 < flip frequency < 0.70	38	63.4%
Systematically stable	Flip frequency ≤ 0.30	11	18.3%

**Table 3:** Performance of MC-PFC on the Chinese blind test set (n=30).

Metric	Value
Accuracy	86.67%
Sensitivity (AD recall)	80.00%
Specificity (HC recall)	90.00%
AUC	0.8710

**Table 4:** Ablation results for calibration strategies (Chinese blind test set, n=30).

#	Configuration	ACC	Sen	Spe	AUC
1	No calibration (direct transfer)	43.33%	70.00%	30.00%	0.5100
2	Distribution shift only	60.00%	70.00%	55.00%	0.5910
3	Polarity flip only (ours)	86.67%	80.00%	90.00%	0.8710
4	Shift + flip	69.33%	48.00%	80.00%	0.7940

**Table 5:** Ablation results for feature space selection.

#	Configuration	AUC
5	ComParE handcrafted features + polarity flip	0.4550
6	Log perplexity (semantic) single stream	0.6200

Note: Configurations 5 and 6 exhibit performance substantially below clinical utility; only AUC is reported as a threshold-independent aggregate metric.

## Data Availability

The ADReSSo 2021 dataset is available through DementiaBank. The NCMMSC2021 dataset is available through the NCMMSC 2021 challenge organizers. The core code implementing the Monte Carlo Polarity Flip Calibration (MC-PFC) method, along with relevant preprocessing scripts, will be made publicly available via a GitHub repository (or provided upon reasonable request to the corresponding author) to support reproducibility and further academic research.

## Abbreviations

The following abbreviations are used in this manuscript:

AD	Alzheimer’s disease
HC	Healthy control
MC-PFC	Monte Carlo Polarity Flip Calibration
AUC	Area under the receiver operating characteristic curve
SVM	Support vector machine
RBF	Radial basis function
LOOCV	Leave-one-out cross-validation
ANOVA	Analysis of variance
MFCCs	Mel-frequency cepstral coefficients
PEFT	Parameter-efficient fine-tuning
CORAL	Correlation Alignment
MMD	Maximum Mean Discrepancy
HuBERT	Hidden-Unit BERT
Wav2Vec	Wave-to-Vector
ADReSSo	Alzheimer’s Disease Recognition through Spontaneous Speech
NCMMSC	National Conference on Man-Machine Speech Communication
ComParE	Computational Paralinguistics Challenge
PET	Positron Emission Tomography
MRI	Magnetic Resonance Imaging
